# Non-invasive prenatal screening for Emanuel syndrome

**DOI:** 10.1186/s13039-020-0476-7

**Published:** 2020-03-04

**Authors:** Yuqin Luo, Jie Lin, Yixi Sun, Yeqing Qian, Liya Wang, Min Chen, Minyue Dong, Fan Jin

**Affiliations:** 1 Department of Reproductive Genetics, Women’s Hospital,School of Medicine, Zhejiang University, 1 Xueshi Road, Hangzhou, 310006 Zhejiang China; 2 Ministry of Education, Key Laboratory of Reproductive Genetics (Zhejiang University), Hangzhou, People’s Republic of China; 3 Centre of Reproductive Medicine, Women’s Hospital, Zhejiang University School of Medicine, Hangzhou, China

**Keywords:** NIPS, Emanuel syndrome (ES), Supernumerary, Microduplication, Translocation, SNP Array

## Abstract

**Objective:**

The aim of this study was to validate the results of two Emanuel syndromes detected by non-invasive prenatal screening (NIPS) screening using invasive methods, providing clinical performance of NIPS on chromosome microduplication detection.

**Methods:**

NIPS was performed to diagnose the Emanuel syndrome. Amniocentesis or cordocentesis was performed to confirm the positive screening result of Emanuel syndrome cases. Fetal sample was detected by karyotyping, fluorescence in situ hybridization (FISH), and single nucleotide polymorphism array (SNP Array). Parental karyotyping and FISH were also carried out.

**Results:**

Two cases with chromosomal abnormalities of 11q23.3q25 and 22q11.1q11.21 were found by NIPS. Chromosomal karyotyping showed that the two fetuses each have a small supernumerary marker chromosome (sSMC), SNP Array further demonstrated double duplications approximately 18 Mb in 11q23.3q25 and 3 Mb in 22q11.1q11.21. FISH confirmed that the small supernumerary marker chromosome (sSMC) was ish der(22)t(11;22) (TUPLE1+, ARSA-). Ultrasound scan and MRI showed some structure malformations in two fetuses. The two mothers were found to be a balanced carrier: 46,XX, t(11;22)(q23.3;q11.2).

**Conclusion:**

NIPS could effectively identify Emanuel syndrome, which may indicate risks of a parent being a balanced rearrangement carrier. The followed confirmation test for positive sample is necessary and ensures the accuracy of the diagnosis.

## What is already known about this topic?

Non-invasive prenatal screening (NIPS) has been validated for common autosomal aneuploidies (trisomies 13, 18, and 21).

NIPS coverage has expanded to detecting certain deletion syndromes such as the 22q11.2 deletion syndrome.

### What does this study add?

We firstly evaluated the potential effectiveness of NIPS to detect the chromosome duplications in Emanuel syndrome fetuses.

NIPS followed by invasive confirmation testing and parental studies would be ideal for the origin determination of fetal duplication and providing accurate counseling.

## Introduction

In the past years, with the rapid development of next-generation sequencing (NGS) and the discovery of cell-free fetal DNA (cfDNA), non-invasive prenatal screening (NIPS) has brought profound changes to detect common autosomal aneuploidies and genetic conditions early in pregnancy using a maternal plasma test [1].The availability of NIPS is obvious, avoiding the risk of miscarriage caused by invasive prenatal diagnostic methods. The clinical validity and utility of NIPS for common autosomal aneuploidies (trisomies 13, 18, and 21) have been endorsed by various clinical guidelines for high risk pregnancies [2]. In a recent meta-analysis in which the results of a large number of studies were pooled, NIPS was found to have a detection rate(DR) of 99.7% for trisomy 21, and a false-positive rate (FPR) of 0.04%.For trisomy 18, the reported figures were 97.9% (DR) and 0.04% (FPR). For trisomy 13, they were 99.0 and 0.04% respectively [3]. However, sub-chromosomal deletion and duplication remain challenging for NIPS owing to the small region of chromosomal abnormality [4]. NIPS coverage has expanded to detecting certain deletion syndromes such as the 22q11.2 deletion syndrome [5], but only few studies have reported the clinical performance of NIPS on duplication detection [6]. Furthermore, there is no evidence to support that NIPS can detect ES, which is a double-segment chromosome duplication of 11q23.3q25 and 22q11.1q11.21.

ES also known as supernumerary der(22)t(11;22) syndrome (OMIM #609029) is a rare chromosomal disorder, which is characterized by multiple congenital anomalies, craniofacial dysmorphism, and significant developmental delay and mental retardation [7].The prevalence of ES is unknown in the general population, and 392 individuals with this condition have been reported (http://ssmc-tl.com/chromosome-22.html). The karyotype of ES, in most cases, is 47,XX,+mar in females and 47,XY, +mar in males. The main cause of ES is a supernumerary marker chromosomes (sSMC) consisting of chromosome 11 attached to chromosome 22. Most ES cases can’t be diagnosed prenatally because of lacking specificity of ultrasound findings and ultrasound abnormalities were only reported in 16% of the patients [7].At present, prenatal diagnosis for ES is still limited to antenatal invasive procedures, such as chorionic villus sampling, amniocentesis or cordocentesis.

In this study, we firstly evaluated the clinical implementation of NIPS to detect the chromosome duplications in ES fetuses. The testing results were successfully confirmed by chromosomal karyotyping, SNP Array, FISH, ultrasound scan and magnetic resonance imaging (MRI). Furthermore, this study aimed to extend NIPS to the genome-wide detection of subchromosomal duplications. NIPS followed by invasive confirmation testing and parental studies would be ideal for the origin determination of fetal duplication and providing accurate counseling.

## Material and methods

### Patient

A 28-year-old G3P1 gravida (patient1) was referred to our hospital for NIPS at 20 weeks’ gestation because of a medial risk for Down syndrome, another 35-year-old G4P1 gravida (patient 2) was offered NIPS at 16 weeks’ gestation for advanced maternal age. To confirm the results, cordocentesis (patient 1) was conducted at 27^+ 6^ weeks’ of gestational age, amniocentesis (case 2) was performed at 19^+ 5^ weeks’ of gestational age. Fetal sample was detected by karyotyping, FISH, and SNP Array. Parental karyotyping and FISH were also conducted to determine the origination of fetal duplications.

Ethical approval for this study was obtained through the Ethics Committee of Women’s Hospital, School of Medicine Zhejiang University. A written informed consent was obtained from all participants in this study.

### NIPS

5 ml of maternal peripheral blood sample was collected into EDTA tube. Maternal plasma was centrifuged at 1600 g for 10 min at 4 °C, followed by transferred to microcentrifuge tubes and centrifuged at 16,000 g for another 10 min at 4 °C. Cell-free plasma was stored at − 80 °C for NIPS. All subsequent standard procedures, library construction, quality control, and multiplexing for sequencing were performed in the joint laboratory of Women’s Hospital, School of Medicine, Zhejiang University and BGI-Shenzhen, China, as described before [8, 9].For non-invasive aneuploidy screening, ten libraries were pooled and sequenced with 35-cycles sequencing using BGISEQ-100 platforms. A pipeline for Fetal Copy Number Analysis through Maternal Plasma Sequencing (FCAPS), was used for sub chromosomal deletion and duplication, which combined with a local weighted polynomial regression-based GC correction strategy, binary segmentation algorithm and dynamic threshold for signal filtering [10].

### Chromosomal karyotyping

Peripheral blood and cordocentesis samples were cultured with Cell Preservation Medium (Sinochrome, Shanghai, China) and fetal amniotic fluid cells were stored at a 5% CO2 incubator at 37 °C,respectively. Cell culture and routine G-banded karyotyping were performed following standard procedures at approximately 350–450 band level.

### FISH analysis

The metaphase chromosomes were analyzed by double-FISH with the 22q11.2 LIS TUPLE1 probe (LSI TUPLE1, Spectrum Orange, Vysis) and the 22q13 LSI ARSA probe (LSI ARSA Spectrum Green, Vysis) on peripheral blood, cordocentesis and amniocentesis samples. Probe hybridization and detection were performed followed the manufacturer’s instructions (Vysis, Downers Grove, IL). The slides were examined by a Zeiss Imager A2 microscope (Zeiss, Germany) and FISH Imaging System Isis (MetaSystems, Germany).

### Chromosome microarray

Genomic DNAs were extracted from amniotic fluid and cord blood sample using the Gentra Puregene Kit (Qiagen, Hilden, Germany) according to the manufacturer’s instruction. Copy number analysis was performed with CytoScan^TM^ HD array platform (Affymetrix, Santa Clara, CA) which contains more than 2,600,000 copy number variations (CNV) markers, including 750,000 genotype-able SNP probes and 1,900,000 non-polymorphism CNV probes followed the manufacturer’s protocols. All data was visualized and analyzed with the Chromosome Analysis Suite (ChAS) software (Affymetrix, USA). The reporting threshold of the copy number result was set at 500 kb for gains and 200 kb for losses.

### Bioinformatics

To understand the aberrations better, we evaluated the duplicated region with our in-house database and the following public databases were used: the Database of Genomic Variants (DGV, http://dgv.tcag.ca/dgv/app/home), the DECIPHER Database (http://decipher.sanger.ac.uk), the PubMed (http://www.ncbi.nlm.nih.gov/pubmed), and the Online Mendelian Inheritance in Man database (OMIM, http://omim.org/), the UCSC (http://genome.ucsc.edu/, hg19) [11].

## Results

### Detection of sub chromosomal copy number aberrations for case 1 and case 2

**Fig. 1:**
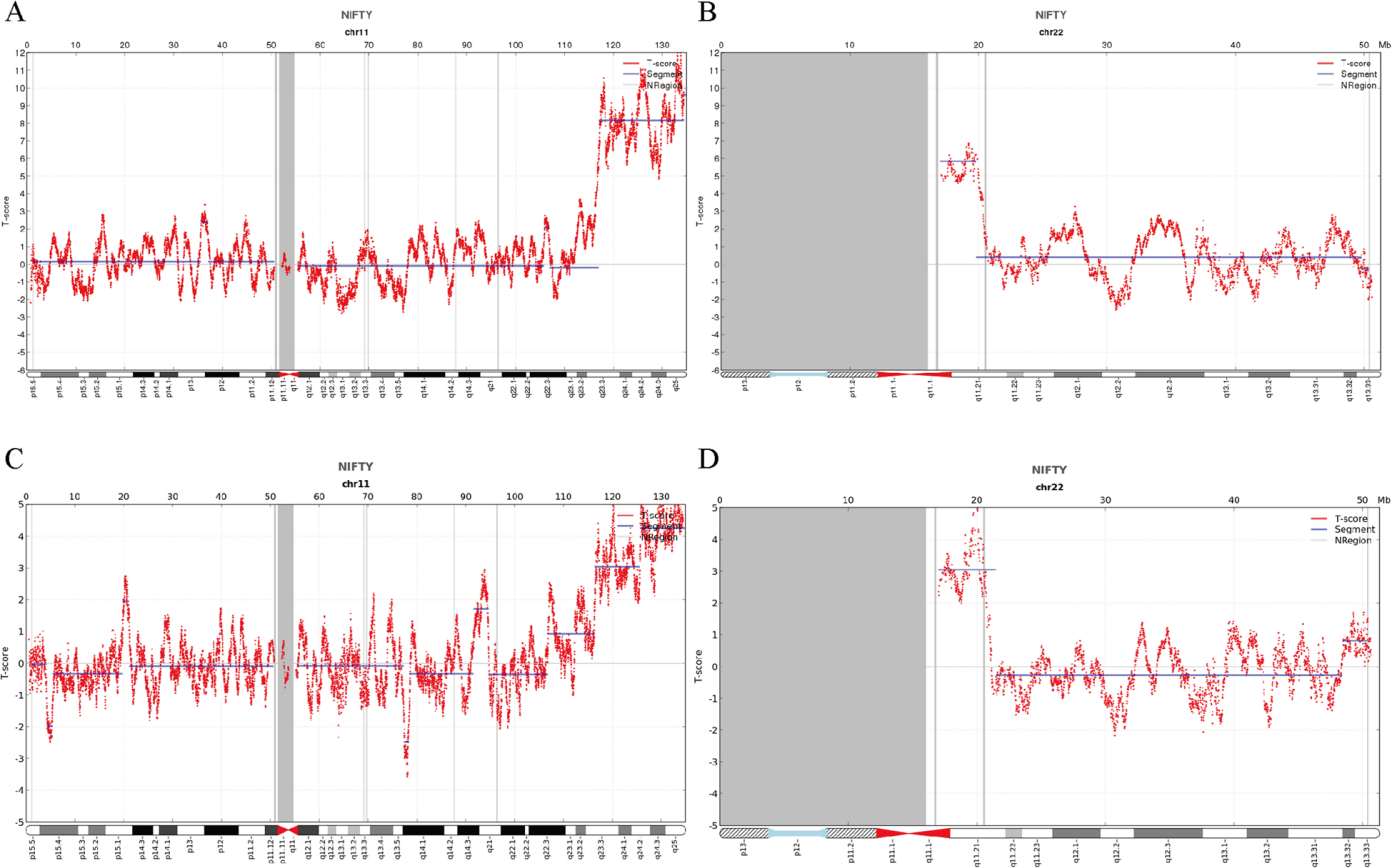
Non-invasive prenatal screening (NIPS) results of two fetuses. (**a** and **b**) NIPS revealed double segmental duplications involving 11q23.3q25 (T-score = 7.856) and 22q11.1q11.21 (T-score = 5.53) of the Case 1 fetus. (**c** and **d**) NIPS report of Case 2 fetus showed abnormal duplication of chromosomes 11q23.3q25 (T-score = 4.121)) and 22 q11.1q11.21 (T-score = 3.212). The horizontal axis represents genomic location (Mb) and the vertical axis represents T-score

The fetal DNA concentration was estimated as 20.052% (case 1) and 7.9%(case 2), the total mapped perfected sequencing reads was 9.354 million (case 1) and 12.661 million (case 2). The cfDNA screening showed that all chromosomes including chromosomes 13, 18 and 21 were normal except chromosomes 11 and 22. The cfDNA test results (case 1) provided the first line of evidence for fetal duplication of chromosomes involving chromosome 11q23.3q25 (T-score = 7.856) and 22 q11.1q11.21 (T-score = 5.53) (Fig.1a- b). NIPS report of pregnant woman (case 2) also showed duplication of chromosomes 11q23.3q25 T-score = 4.121) and 22 q11.1q11.21 (T-score = 3.212) (Fig.1c-d).

### Chromosome microarray for case 1 and case 2

**Fig. 2:**
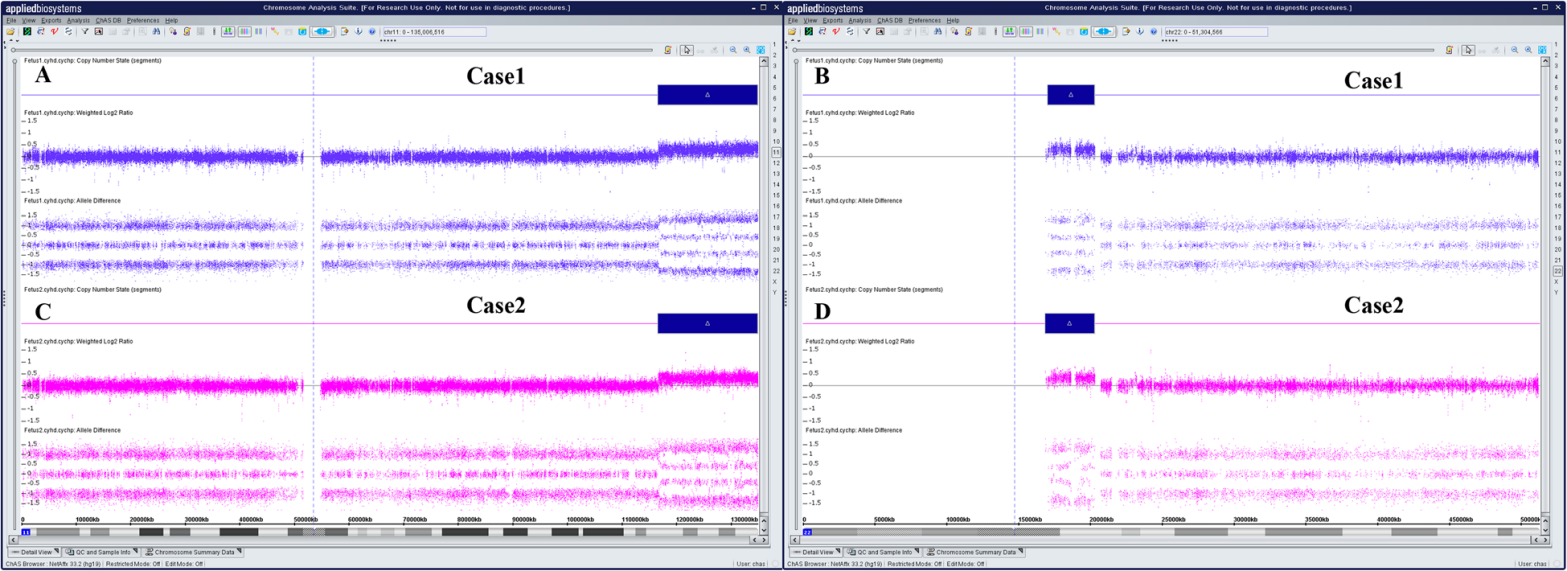
CMA confirmed the duplication. (**a**) CMA for fetus of case 1 reveals approximately 18.2 Mb duplication in chromosome 11q23.3q25 (chr11:116,696,886-134,938,470).(**b**) CMA for fetus of case 1 reveals 3.2 Mb duplication in chromosome 22q11.1q11.21 chr 22:17,055,733-20,311,858).(**c**) In case 2, an approximately 18.2 Mb duplication in chromosome11q23.3q25(chr11:116,681,007-134,938,470) .(**d**) In case 2, 3.4 Mb duplication in chromosome 22q11.1q11.21. The horizontal axis represents genomic location (Mb). The blue rectangle represents the duplication

CMA for fetus of case 1 reveals approximately 18.2 Mb duplication in chromosome 11q23.3q25 (chr11:116,696,886-134,938,470) (Fig. 2a) and 3.2 Mb duplication in chromosome 22q11.1q11.21(chr 22:17,055,733-20,311,858) (Fig. 2b). In case 2, an approximately 18.2 Mb duplication in chromosome11q23.3q25(chr11:116,681,007-134,938,470) (Fig. 2c) and 3.4 Mb duplication in chromosome 22q11.1q11.21(chr22:16,888,899-20,311,858) (Fig. 2d).

### Karyotyping and FISH verification for case 1 and case 2

**Fig. 3:**
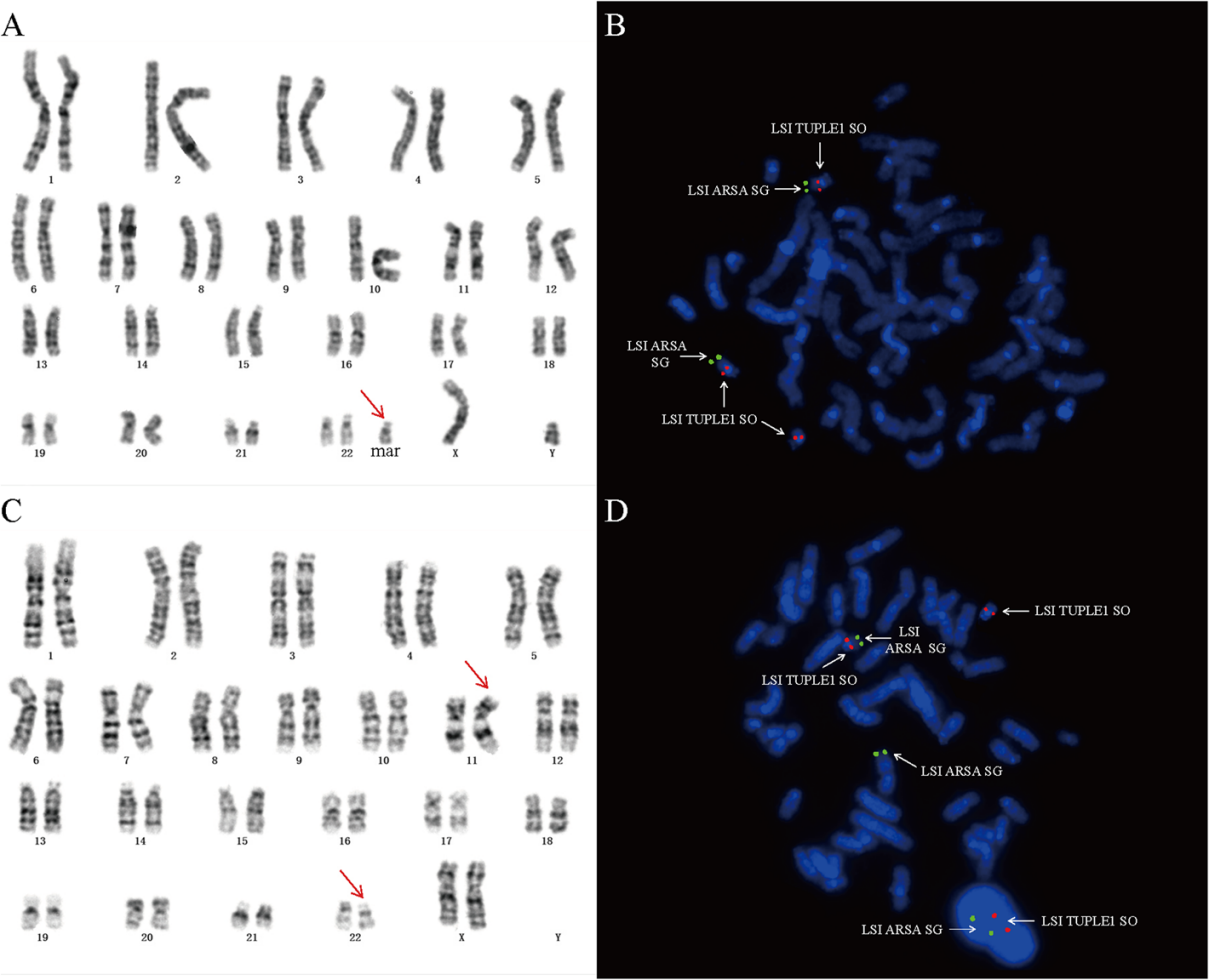
Karyotype and FISH analysis on the fetus and the mother of case 1 (**a**) Karyotype of fetus: 47,XY,+der(22)t(11;22). (**b**) FISH image of the fetus with 47,XY,+der(22)t(11;22) (TUPLE1+, ARSA-),green signals the 22q13 LSI ARSA and red signals 22q11.2 LIS TUPLE1.(**c**) Karyotype of the mother:46,XX,t(11;22)(q23.3;q11.2). (**d**) FISH image of the mother with t(11;22)(q23.3;q11.2),green signals the 22q13 LSI ARSA and red signals 22q11.2 LIS TUPLE1

The fetal karyotype (case 1) showed an extra small marker chromosome, and the fetal karyotype was 47,XY,+mar (Fig. 3a). Meterphase FISH analysis with the 22q11.2 LIS TUPLE1 and 22q13 LSI ARSA probe and further revealed a TUPLE1duplication (Fig. 3b). Karyotyping of fetal parents were also performed. The mother was found to be a balanced carrier: 46,XX,t(11;22)(q23.3;q11.2) (Fig. 3c). The result of FISH verified the translocation of karyotype (Fig. 3d). The final fetal karyotype based on FISH and CMA was 47,XY, +der (22) mat.ish der (22)(TUPLE1+, ARSA-). arr [hg19] 11q23.3q25(116,696,886-134,938,470)× 3,22q11.1q11.21(17,055,733-20,311,858)× 3. The fetal karyotype (case 2) was 47,XX,+mar, the final fetal karyotype of case 2 was 47,XX,+der(22) mat. ish der (22)( TUPLE1+, ARSA-). arr[hg19] 11q23.3q25(116,681,007-134,938,470)x3,22q11.1q11.21(16,888,899-20,311,858)x3.

### Ultrasound scan and MRI

**Fig. 4:**
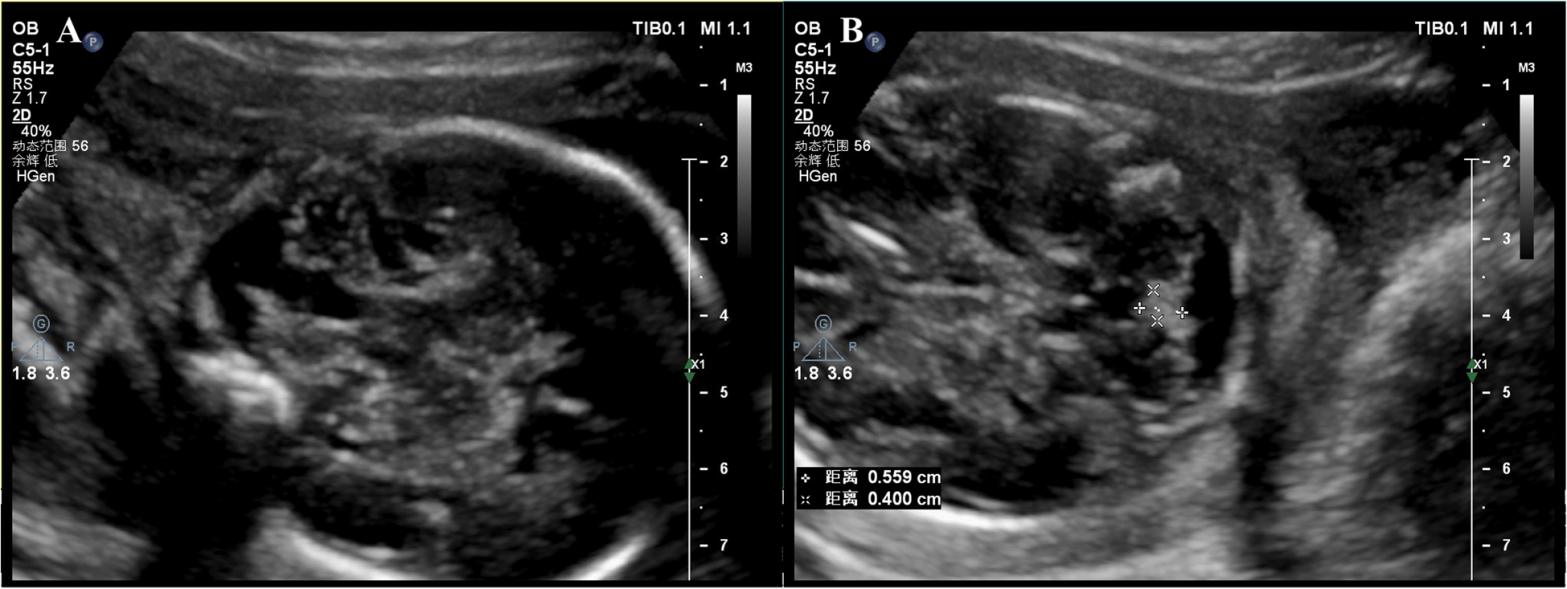
Ultrasonographic findings of the Case 1 fetus. The fetus showed various findings including (**a**) inferior vermian hypoplasia (**b**) Posterior fossa pool width: 0.7 cm

Ultrasound and MRI scan were carried out and showed some malformations of the Case 1 fetus. Fetal ultrasonography showed hypoplasia of the cerebellar vermis and was characterized by an normal posterior fossa (Fig. 4) at 26 weeks of gestational age. MRI showed inferior vermian hypoplasia and a normal posterior fossa cyst communicating, which was similar with Dandy–Walker malformation (DWM). Case 2 fetal had a measured NT of 2 mm.

## Discussion

ES is an inherited chromosomal abnormality syndrome which usually results from a 3:1 meiotic disjunction in the carrier of a t(11;22)(q23;q11.2) translocation [12]. The two patients with ES in this study were inherited from maternal balanced translocation carrier. The two mothers have no clinical symptoms. ES is characterized by distinctive phenotype, severe intellectual disability, multiple congenital anomalies, features including microcephaly, cleft or high-arched palate, ear pits, preauricular tag or sinus, micrognathia, kidney abnormalities, heart defects, and cryptorchidism [7, 13]. Unfortunately, most of ES cases haven’t been diagnosed during pregnancy. Previous study of 63 ES patients has shown that only 16% of patients during pregnancy can be detected by ultrasound [7]. Ultrasound findings includes ear pits, micrognathia, heart malformations, cleft palate (most commonly an atrial-septal defect) and Dandy-Walker malformation. However, Dandy-Walker malformation in association with ES has rarely been diagnosed prenatally in the literature [14, 15]. Here, we present the forth case of Dandy-Walker malformation in association with ES that has been detected prenatally, which may be an associated feature of ES. It is obviously not a diagnostic marker due to lacking specificity and sensitivity. The population-based prenatal screening of ES could be difficult because there is no practical, specific, and sensitive method to diagnose ES. Therefore, it is necessary to develop a highly accurate noninvasive genetic test. The patients with ES in present study are the first, to the best of our knowledge, to be detected by NIPS.

With the development of using whole-genome sequencing of cfDNA in maternal plasma, the performance of cfDNA screening in detecting fetal aneuploidies was well demonstrated [16]. The clinical utility of NIPS have been accepted as a highly effective screening test for common autosomal aneuploidies (trisomies 13, 18, and 21). Its application of deletion and duplication has gradually been proved, although the sensitivity and specificity is somehow lower than those for common chromosome aneuploidies. More recently, this methodology has been applied successfully to the detection of certain microdeletion syndromes, such as Prader-Willi/Angelman syndrome, Cri-du-chat syndrome, DiGeorge syndrome/ velocardiofacial syndrome [17]. However, NIPS efficacy of detecting microduplications is still challenging because there is only a 1.5-fold change in copy number (3:2) in place of a two-fold change (1:2) during microdeletions. In this study, we adopted the T-score algorithm and the FCAPS method to identified successfully two microduplications involving chromosomes 11q23 and 22q11.2, even those as small as 3256 kb, and the results were further verified with conventional karyotyping, FISH and CMA in our diagnosis. To the best of our knowledge, this is the first study to screen the ES by NIPS. The main advantage of our approach is that the required sequencing reads are only about 10 million, making non-invasive detection of microduplications more realistic in clinical practice. The correct detection of duplication region by NIPS was verified by three different molecular methods, indicating that non-invasive screening can be applied to screen for ES. This is a proof of concept study with potential clinical implementation in NIPS. For many other types of microduplications, currently NIPS has stilled been doubted in application not only because of the uncertain efficacy of NIPS impacted by low-coverage, fetal fraction and size of microduplication, but also of the difficulties to consulting even the microduplications were confirmed by other techniques, since the clinical variable phenotype of microduplications varies from no symptoms to severe symptoms.

For couple considering a possible termination of pregnancy, invasive prenatal tests remain the gold standard of genetic diagnosis because multiple factors such as confined placental mosaicism [18], maternal mosaicism [19], a vanishing twin [20] or maternal malignancy [21] may affect the accuracy of cfDNA screening. Verification of positive NIPS results is required. Clinicians should offer integrated genetic counseling using molecular genetic testing consisting of karyotyping analysis, FISH and CMA, as shown in this case. The 11q23.3q25 duplication can be evaluated using database such as OMIM and DECIPHER. 11q23.3q25 duplication may be associated with hypophrenia, cardiovascular disease or dysplasia. Another duplication region 22q11.1q11.21 (3256 kb or 3423 kb) constitute the 22q11 microduplication syndrome. The 22q11.21 microduplication are reported to share several characteristic features including dysmorphic facial features, congenital heart disease, velopharyngeal insufficiency, urogenital abnormalities, and immunologic defects [22]. Fetuses with ES caused by maternal balanced translocation were initially indicated by NIPS and confirmed by CMA. The determination of maternal origin was crucial in assessing recurrence risks in the future reproductive choice for couples. It is well-known that carriers of balanced reciprocal translocations have increased risks of unbalanced gametes [23], resulting in infertility, recurrent miscarriage, and children with abnormalities. Fortunately, these couples can turn to preimplatation genetic diagnosis (PGD) or prenatal diagnosis to avoid fetus with ES for future pregnancies. There has been success with PGD in the carrier of a t(11;22)(q23;q11.2) translocation [24].

## Conclusions

We report here two fetuses with ES determined by NIPS and confirmed by invasive diagnosis. This is a proof of concept study with potential clinical implementation in NIPS. The ES diagnosis strategy used in this study showed high accuracy and could be applied to ES screening. The main advantage of our approach is the low coverage sequencing requires only 10 million reads, making NIPS more practical in clinical practice.

## Supplementary information

**Additional file 1: Figure S1.** Karyotype and FISH analysis on the fetus of case 2 (A) Karyotype of fetus: 47,XX,+der(22)t(11;22). (B) FISH image of the fetus with 47,XX,+der(22)t(11;22) (TUPLE1+, ARSA-),green signals the 22q13 LSI ARSA and red signals 22q11.2 LIS TUPLE1.

## Data Availability

The data supporting the conclusions of this article is included within the article.
