# Supplementary figures and images for: Non-invasive prenatal screening for Emanuel syndrome
Source: Mol Cytogenet. 2020 Mar 4;13:9. doi: 10.1186/s13039-020-0476-7 (PMC7057502; doi:10.1186/s13039-020-0476-7)

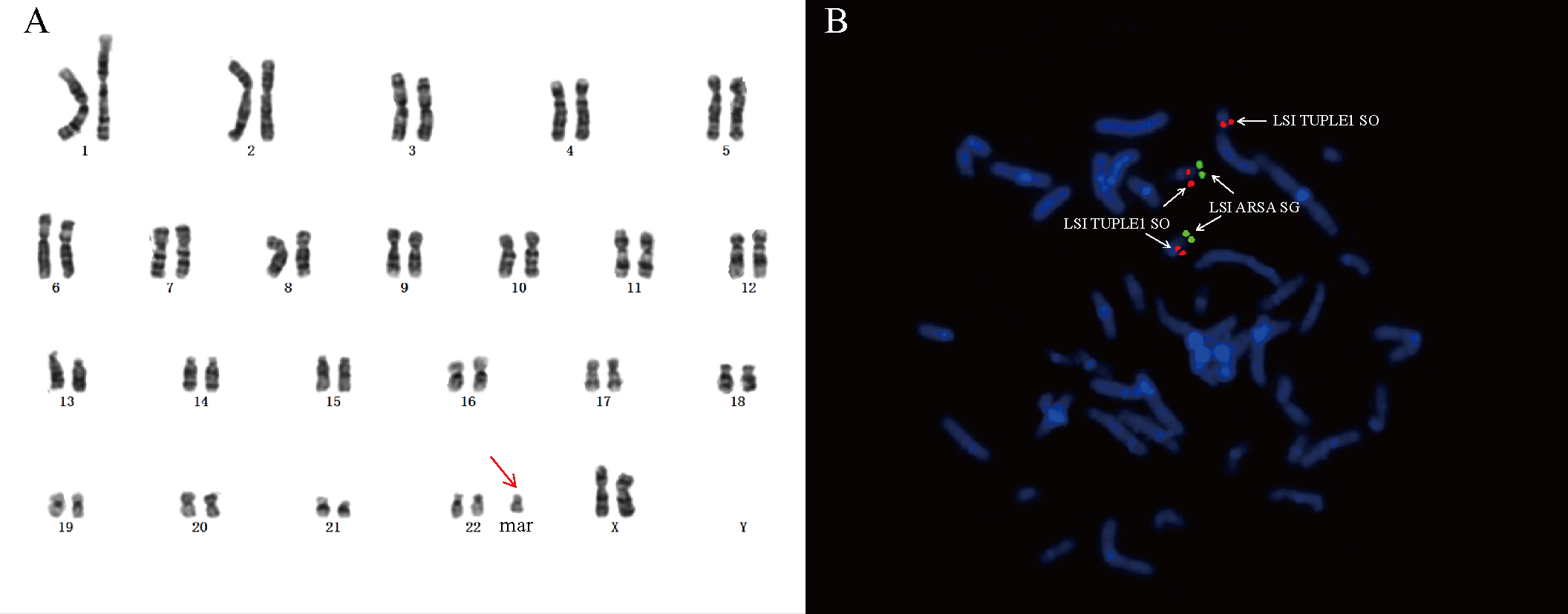

Supplement: Supplementary file 1 — Additional file 1: Figure S1. Karyotype and FISH analysis on the fetus of case 2 (A) Karyotype of fetus: 47,XX,+der(22)t(11;22). (B) FISH image of the fetus with 47,XX,+der(22)t(11;22) (TUPLE1+, ARSA-),green signals the 22q13 LSI ARSA and red signals 22q11.2 LIS TUPLE1. [file 13039_2020_476_MOESM1_ESM.tif]
